# Clinical and Procedural Outcomes of 5-French versus 6-French Sheaths in Transradial Coronary Interventions

**DOI:** 10.1097/MD.0000000000002170

**Published:** 2015-12-31

**Authors:** Alberto Polimeni, Francesco Passafaro, Salvatore De Rosa, Sabato Sorrentino, Daniele Torella, Carmen Spaccarotella, Annalisa Mongiardo, Ciro Indolfi

**Affiliations:** From the Division of Cardiology, Department of Medical and Surgical Sciences, “Magna Graecia” University (AP, FP, SDR, SS, DT, CS, AM, CI); and URT-CNR, Department of Medicine, Consiglio Nazionale delle Ricerche, Catanzaro, Italy (CI).

## Abstract

The radial artery has been increasingly used for its favorable safety profile. However, no conclusive data are available on the optimal sheath size. In particular, it is seemingly difficult to weight both advantages and disadvantages of narrower versus larger sheaths size. Despite several studies were performed to compare the use of 6-Fr to the smaller 5-Fr sheaths, these were mostly small, single center-studies, yielding various results.

We performed a comprehensive meta-analysis of all available studies comparing the use of 5-Fr versus 6-Fr sheaths in coronary procedures through the TRA.

Studies comparing a 5-Fr versus a 6-Fr sheaths were searched for in PubMed, the Cochrane Library, and ISI Web of Knowledge databases.

Studies were deemed eligible if they only included patients undergoing transradial cardiac catheterization with 5-Fr or 6-Fr system and reported at least one of these parameters: contrast dye volume, procedural success, procedural time, access complications, radial artery occlusion, and bleedings.

Odds ratio (OR) and the mean difference (MD) were respectively used for dichotomous and continuous variables as summary measures. Both the random-effects model and the fixed effect models were used for computation of meta-analyses. Heterogeneity was assessed by means of the Cochrane *Q* test. Metaregression was calculated using the unrestricted maximal likelihood random effects model.

The use of a 5-Fr system is associated with a significant lower contrast medium administration (MD = −22.20 [−36.43 to −7.96], *P* < 0.01) and significantly reduces bleedings (OR = 0.58 [0.38–0.90], *P* = 0.02), without compromising procedural success (OR = 0.95 [0.53–1.69], *P* = 0.86) or procedure length (OR = 0.55 [−2.58 to 3.69], *P* = 0.73), compared to the 6-Fr system. Despite no significant difference was observed between the groups (OR = 0.88 [0.50–1.56], *P* = 0.67), at metaregression RAO incidence in the 5-Fr group was increasingly lower as the percentage of women included into the study increased (*P* = 0.02).

Some potentially interesting technical details, such as sheath length, hydrophilic coating, or periprocedural anticoagulation, were not homogeneously reported in individual studies.

Results of the present meta-analysis confirm the excellent safety profile of transradial procedures both with 5-Fr and 6-Fr system. A 5-Fr system could be preferred in patients with a higher bleeding propensity or kidney injury.

## INTRODUCTION

The radial artery has been increasingly used as the preferred access site for coronary procedures because of lower rates of access site complications, shorter hospital stay, and improved patient comfort, compared with the transfemoral access.^1,2^

The substantial benefits over the transfemoral approach (TFA) drove the progressive adoption of the transradial approach (TRA) worldwide, especially after it was shown to be safe, even during the implementation phase.^3^

Despite the current widespread use of the TRA, no conclusive data are available on the optimal sheath size. If on one hand, some operators prefer a 5-Fr system to minimize the risk for spasm or access site bleedings, 6-Fr sheaths are widely used since they allow the use of a larger selection of devices and techniques.^4^ Despite several studies were performed to compare the use of 6-Fr to the smaller 5-Fr sheaths,^5–7^ they are mostly single center-based and included a limited number of cases, which strongly limit their discrimination potential.

For this reason, we performed a comprehensive meta-analysis of all available studies comparing the use of 5-Fr versus 6-Fr sheaths in coronary procedures performed through the TRA.

## METHODS

### Search Strategy and Study Selection

Published trials comparing 5-Fr versus 6-Fr sheaths were searched for in PubMed, the Cochrane Library, and ISI Web of Knowledge electronic databases up to January 16, 2015. The following search syntax was used: (“transradial” or “radial”) and (“sheath” or “catheter”). Time of publication was not a limiting criterium for our analysis. Only articles reported in English were included. All reports, including the search terms, were independently screened by 2 independent investigators (AP, FP) for relevance and eligibility. Additionally, references from relevant articles were also scanned for eligible studies. The authors discussed their evaluation and any disagreement was resolved through discussion and re-reading. All selected studies were thoroughly checked and classified by the author's institution in order to avoid any bias from duplicity of data.

Studies were deemed eligible if they only included patients undergoing transradial cardiac catheterization with 5-Fr or 6-Fr sheaths and reported at least one of these parameters: contrast dye volume, procedural success, procedural time, radial artery occlusion, and bleedings. Exclusion criteria were (just 1 was sufficient for study exclusion) duplicate publication, endpoint measure not specified.

### Data Abstraction and Validity Assessment

Baseline characteristics, procedural variables, as well as the number of clinical events were extracted from the single studies, through carefully scanning of the full article by 2 independent reviewers (AP, FP). Divergences were resolved by consensus. In particular, the following data were abstracted: year of publication, site of patient recruitment, number of patients, study design, and baseline patients’ characteristics (Table 2). Quality assessment was performed according to the Prisma guidelines.^8^

**TABLE 2:**
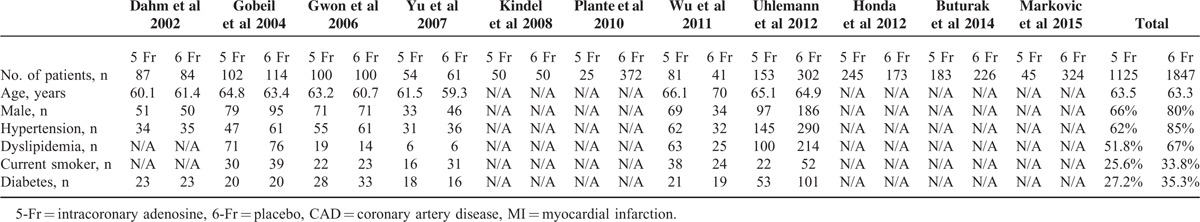
Baseline Patient's Characteristics

### Statistical Analysis

For dichotomous variables the summary measure used was odds ratio (OR) with 95% confidence intervals. For computation of the meta-analysis OR were converted to a logarithmic scale. To facilitate interpretation of the results by the readers, the resulting summary effect (on a log scale) was converted back to OR. For continuous variables the mean difference was used as the summary measure. The random-effects was used to assess the effect of model assumptions on our conclusions.^9^

Heterogeneity was assessed by means of the Cochrane *Q* test using a chi-squared function, with *P* values <0.10 considered significant for heterogeneity as previously described.^10^ Additionally *I*^2^ values were calculated for estimation of variation among studies attributable to heterogeneity. A fixed effect or a random-effects model was used as appropriate, with a 2-sided *P* value <0.05 considered significant. Small study effects were evaluated through graphical inspection of funnel plots and statistically using Egger's test (See Supplementary Figure 1).^11^ Metaregression analyses were calculated using the unrestricted maximal likelihood random effects model, as already previously described.^10^ Analyses were performed by means of Excel spreadsheets and Review Manager 5.3.

All analyses were based on previous published studies; thus, no ethical approval and patient consent are required.

## RESULTS

### Study Search, Selection, and Baseline Evaluation

**FIGURE 1:**
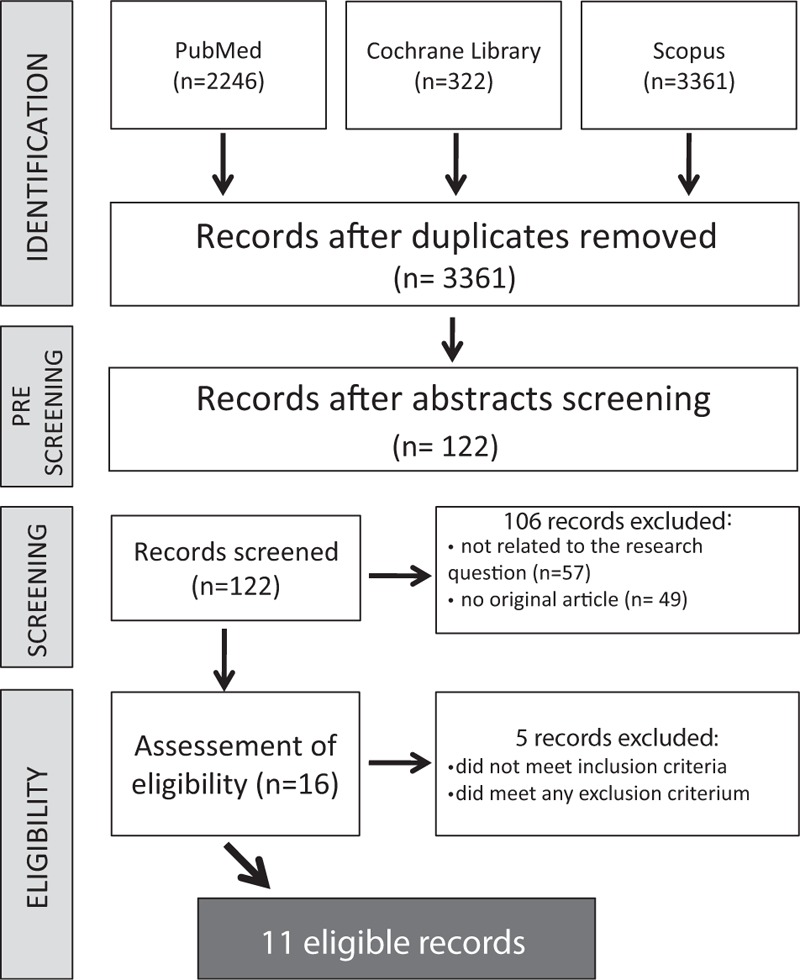
Flowchart of study search, screening, and selection.

Our database search retrieved a total of 3361 studies after removal of duplicates, which were reduced to 122 studies after an initial prescreening. Hundred and six studies were then excluded for one of the following reasons: (a) they were not related to our research question; (b) they were not original articles. The remaining articles were checked through their full text, resulting in further exclusion of 5 studies, which did not meet our prespecified inclusion criteria.^12–16^ Finally, a total of 11 studies were available for the analysis, 3 randomized studies^5–7^ and 8 nonrandomized studies.^17–24^ The study selection procedure is reported in detail in Figure 1, whereas Table 1 summarizes the most relevant characteristics of the selected studies. Clinical characteristics of the study population are summarized in Table 2.

**TABLE 1:**
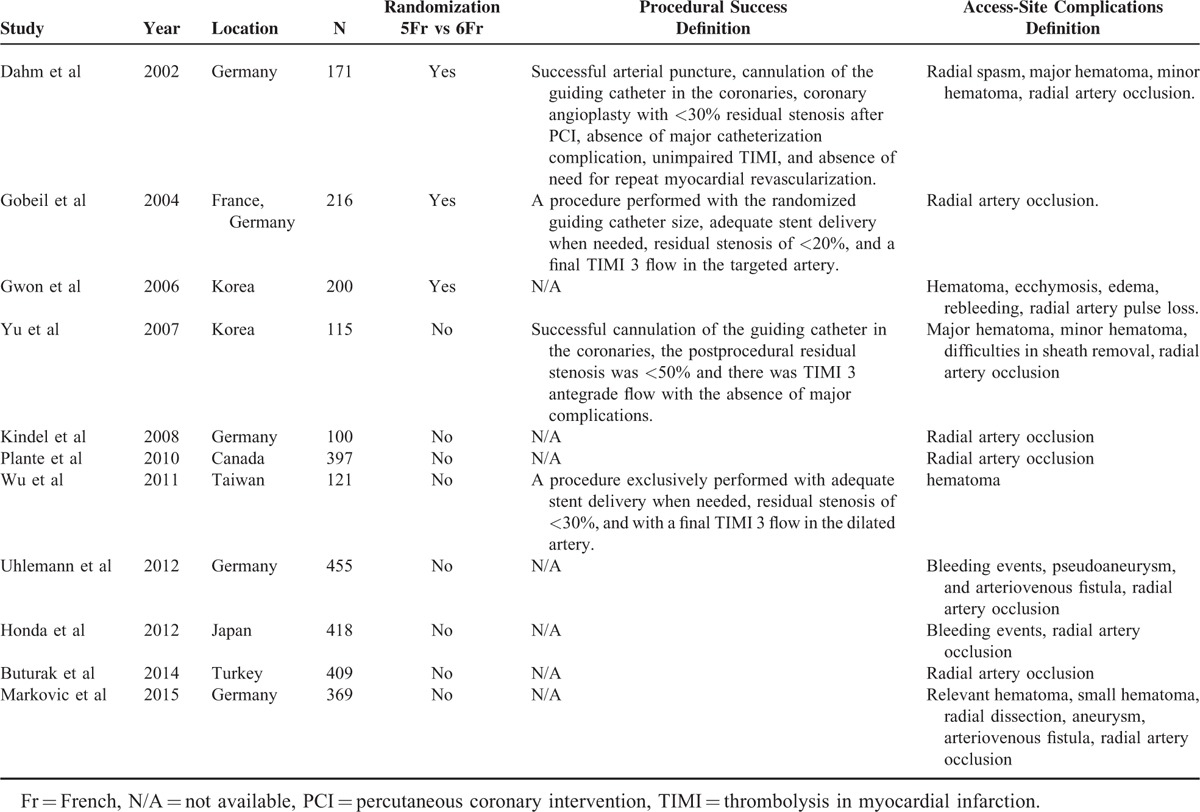
Characteristics and Endpoint Definition of Studies Included in the Meta-Analysis

### Impact of 5-Fr Sheaths on Procedural Performance

The procedural success rate and mean procedural time and the mean amount of contrast medium administered during the procedure were analyzed separately.

**FIGURE 2:**
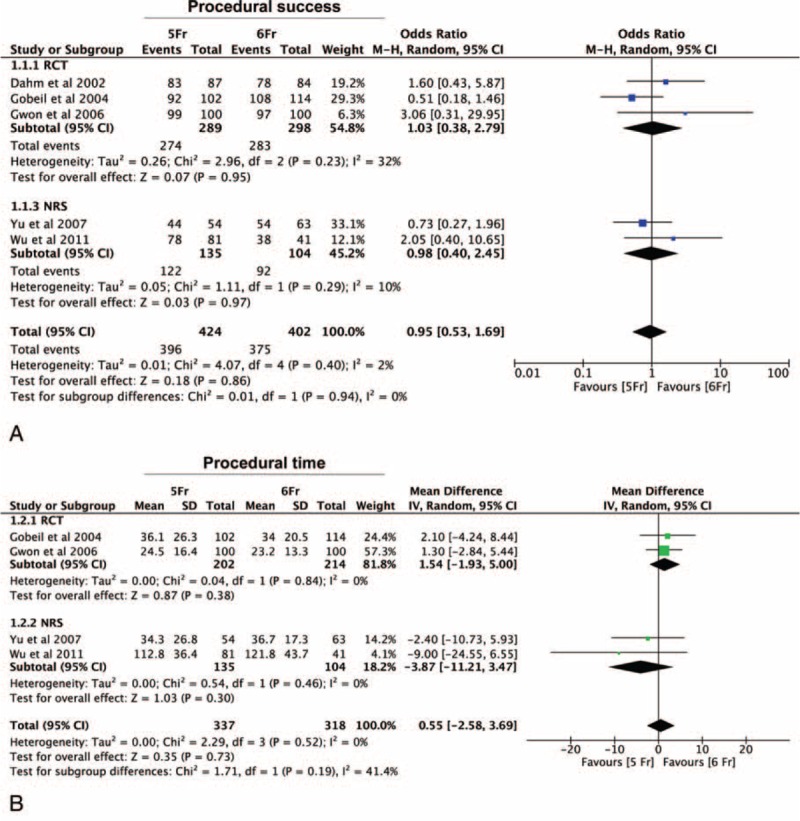
Impact of 5-Fr catheter on procedural performance. Panel A—meta-analysis of difference in procedural success showing no statistically significant difference in procedural success between 5-Fr and 6-Fr arms (*P* = 0.86). Panel B—meta-analysis of difference in procedural time between 5-Fr and 6-Fr arms demonstrating no significant differences in procedural time between the groups (*P* = 0.73). Procedural time (minutes) is reported as mean for each study arm.

A total of 5 studies reported the incidence of procedural success, including 826 procedures. We found no evidence of funnel plot asymmetry for any of the analyzed studies (See Supplementary Figure 1, *P* > 0.10). No statistically significant difference in the rate of procedural success (OR = 0.95 [0.53–1.69], *P* = 0.86) was observed between 5-Fr versus 6-Fr arms (Figure 2A). As both randomized (RCT) and nonrandomized studies (NRS) were included in this analysis, we performed a subgroup analysis, revealing a similar result for both study subgroups.

In addition, analyzing the 4 studies reporting results on mean procedural time, no significant differences were observed between the study arms (OR = 0.55 [−2.58 to 3.69], *P* = 0.73) (Figure 2B). Of note, a similar result was observed in the RCTs and the NRS subgroups, despite the minimal, nonsignificant, numerical difference.

**FIGURE 3:**
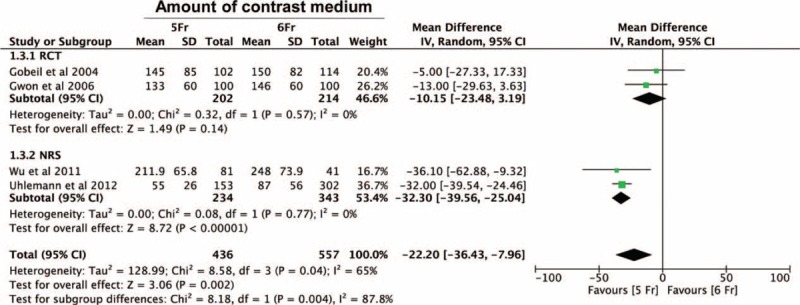
Meta-analysis of standardized mean difference in the amount of contrast medium administrated during the procedure showing a statistically significant difference between the groups in favor of the 5-Fr arm (*P* < 0.01). RCT = randomized controlled trial; NRS = nonrandomized study.

Interestingly, a statistically significant difference in the amount of contrast medium administrated during the procedure was found in favor of 5-Fr group (MD = −22.20 [−36.43 to −7.96], *P* < 0.01), corresponding to a saving of ∼22.2 mL of contrast medium per procedure (Figure 3). Although the same trend was observed both in the RCT- and the nonrandomized subgroups, this difference was statistically significant only within the nonrandomized subgroup (MD = −32.30 [−39.56 to −25.04], *P* < 0.01).

### Impact of 5-Fr Sheaths on Access-Site Complications

**FIGURE 4:**
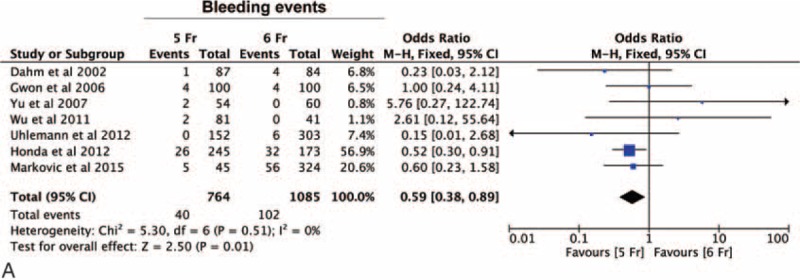
Impact of 5-Fr catheter on bleeding events. Panel A—meta-analysis of the difference in the incidence of bleedings between the groups demonstrating a significant reduction of bleeding events in the 5-Fr arm (*P* = 0.02).

Seven studies reported the incidence of bleedings events, for a total of 1849 procedures. Comparing the incidence of bleedings between the study arms, we found a significant reduction in the incidence of bleeding events in the 5-Fr arm (OR = 0.58 [0.38–0.90], *P* = 0.02) (Figure 4A). Although the same trend was observed both in the RCT- and the NRS subgroups, this difference was statistically significant only for the NRS subgroup (OR = 0.58 [0.35 – 0.94], *P* = 0.03). Interestingly, at metaregression analysis, this advantage was progressively lost in those studies with a higher percentage of PCI performed in addition to the diagnostic angiography (*P* = 0.01) (Figure 4B).

**FIGURE 5:**
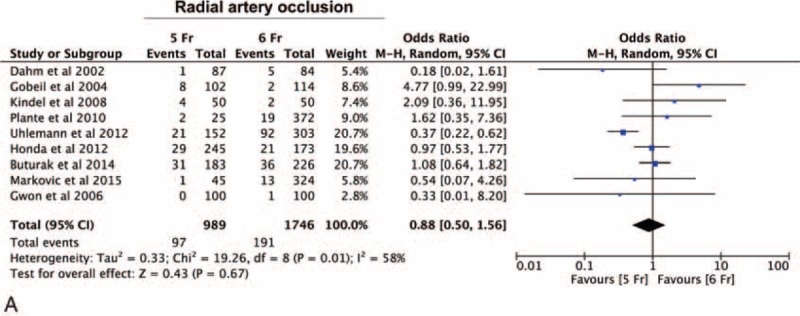
Impact of 5-Fr catheter on radial artery occlusion (RAO). Panel A—meta-analysis of the difference in the incidence of radial artery occlusion between the 5-Fr and 6-Fr arms showing no statistically significant difference in RAO incidence between the arms (*P* = 0.67).RAO = radial artery occlusion.

Finally, we evaluated the incidence of radial artery occlusion (RAO). Nine studies reported the incidence of an RAO, for a total of 2735 procedures. No statistically significant difference was observed in RAO incidence (OR = 0.88 [0.50–1.56], *P* = 0.67) between the 5-Fr and the 6-Fr arms (Figure 5A). Subgroup analysis did not disclose any substantial difference between the RCT and the NRS study subgroups. However, at metaregression an increasing benefit was evident with the 5-Fr sheaths as the percentage of women included into the study increased (*P* = 0.02) (Figure 5B).

### Metaregression Analysis

**FIGURE 6:**
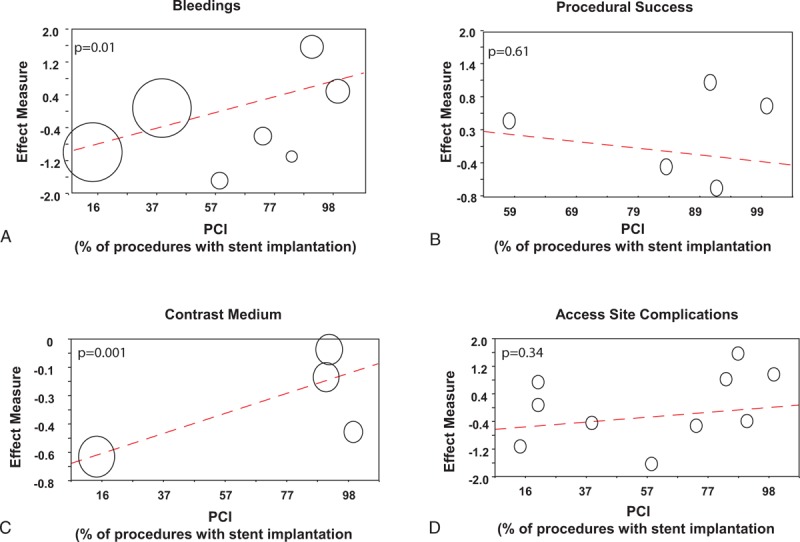
Metaregression analyses on the influence of PCI on bleeding events, procedural success, contrast medium administration, and access site complications. Panel A—metaregression analysis on the influence of PCI on bleeding events. A significant correlation is evident from the graph (*P* = 0.01), indicating that the benefit on bleedings observed with the 5 French system is progressively lost in those studies with a higher percentage of PCI performed in addition to the diagnostic angiography. Largeness of circles represents the weight of each individual study. Panel B—metaregression analysis on the influence of PCI on procedural success. A not significant correlation is evident from the graph (*P* = 0.61). Panel C—metaregression analysis on the influence of PCI on contrast medium administration. A significant correlation is evident from the graph (*P* = 0.001). Panel D—meta-regression analysis on the influence of PCI on access site complications. A not significant correlation is evident from the graph (*P* = 0.34). PCI = percutaneous coronary intervention.

Given the potential differences between diagnostic and interventional procedures, we used the percentage of interventional procedures in every single study as a moderator in a metaregression analysis with the effect size of all endpoints evaluated. Consequently, we used this information—namely the percentage of interventional procedures in single studies as to moderator variable to compute a metaregression analysis. Interestingly, we found a significant interaction across the studies between sheaths caliber and the percentage of interventional procedures on the incidence of bleedings (*P* = 0.01) and on the amount of contrast medium administered (*P* = 0.001), whereas no significant interaction was observed for procedural success (*P* = 0.61) or access site complications (*P* = 0.34). Results of metaregression analyses with percentage of interventional procedures are displayed in Figure 6.

Furthermore, as local access site complications are often more frequent in women than in men, we evaluated the interaction between the effect size and the percentage of women included in single studies by means of metaregression analysis. Interestingly, we found a significant interaction across the studies between sheaths caliber and the percentage of female patients included in single studies on the rate of RAO (*P* = 0.02), whereas no significant interaction was observed for procedural success (*P* = 0.87), amount of contrast medium administered (*P* = 0.70), or total access site complications (*P* = 0.06).

**FIGURE 7:**
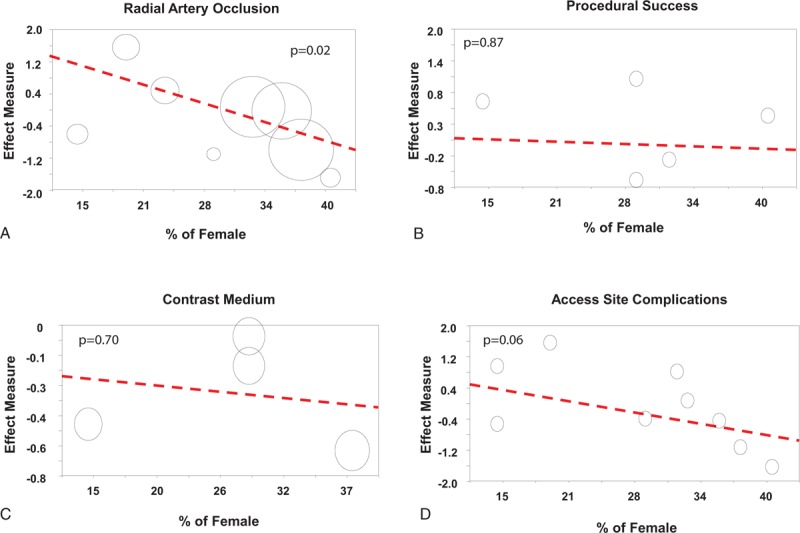
Metaregression analyses on the influence of female sex on radial artery occlusion, procedural success, contrast medium administration, and access site complications. Panel A—metaregression analysis on the influence of female sex on radial artery occlusion. A significant correlation is evident from the graph (*P* = 0.02), indicating an increasing benefit with the 5-Fr sheath as the percentage of women included into the study increased. Largeness of circles represents the weight of each individual study. Panel B—metaregression analysis on the influence of female sex on procedural success. A not significant correlation is evident from the graph (*P* = 0.87). Panel C—metaregression analysis on the influence of female sex on contrast medium administration. A not significant correlation is evident from the graph (*P* = 0.70). Panel D—meta-regression analysis on the influence of female sex on access site complications. A not significant correlation is evident from the graph (*P* = 0.06).

Results of metaregression analyses with percentage of female patients included are displayed in Figure 7.

## DISCUSSION

The major findings of the present meta-analysis are: (a) the total amount of contrast medium necessary to complete the procedure are significantly lower using a 5 French approach, compared to the 6 French; (b) significantly less access-site bleedings were observed with the 5-Fr approach; (c) the benefits observed with the 5 French approach did not affect the procedural success rate, which was virtually comparable between the arms; (d) although no frank evidence of a lower post-procedural radial occlusion rate was observed in the 5-Fr arm, a significant benefit was evident at metaregression, with a larger effect in the studies with a higher percentage of women.

The present meta-analysis is the first comparing the procedural success with the 5 French versus the 6 French transradial approach. Our findings, based on 3 randomized and 8 prospective nonrandomized studies, are of large interest for interventional cardiologists. In fact, we provide the first demonstration that use of a 5 French system for transradial catheterization is associated to a significant reduction in the amount of contrast medium administered, compared to the 6 French, with relevant implications for patients’ safety. A lower amount of contrast medium is associated to a lower risk of contrast-induced nephropathy (CIN) and, therefore, the 5 French strategy could be considered in patients with kidney failure or when repetitive procedure is needed.^25^

In addition, the novel evidence of a significantly lower bleeding rate with the 5 French also represents an interesting finding, given the impact of bleeding complications on patients’ clinical outcome.^26,27^ In fact, despite it was shown to be safer that the transfemoral access, diffusion of the transradial access did not undergo the expected fast and large acceptance, yet.^3^ In this regard, our results of an even better safety profile with a 5 French system could propel the diffusion of the transradial approach. Interestingly, as demonstrated at metaregression, the benefit on bleedings observed with the 5 French system was actually progressively larger as the number of procedures including a PCI increased in single studies.

Altogether, a lower complication rate—including bleedings and contrast-induced nephropathy—also contributes to reduce the social costs of coronary procedures. In fact, an uneventful hospital stay avoids the adjunctive treatment costs associated to eventual complications, reduces patients’ hospital stay, allowing a quick return of patients to their social duties, as well as a higher patient turnover, in a time where several healthcare systems are facing the issue of long waiting queues for patients.

Given the excellent results with the 5-Fr system, this should be the preferred default choice, especially at the beginning of a radial program. Interestingly, at metaregression the benefit observed in terms of RAO rate was larger as the number of women included into the single studies grew. This could be a consequence of the average thinner radial artery diameter in women, making the use of larger arterial sheaths more traumatic than for men. In fact, 1 potential advantage with the 5-Fr sheet could be the consequent reduction of traumatism to the arterial wall, which is not irrelevant. In fact, a recent study showed that the presence of microdissections at the radial access site, evaluated by means of optical coherence tomography (OCT), is a potent risk factor for radial artery occlusion,^28^ one of the sneakiest complications of the transradial approach, frequently asymptomatic and underdiagnosed, but not a clinically irrelevant complication.^29,30^

However, it should be pointed out that a learning curve is necessary for successful use of the 5 French strategy, to get familiar with the different trackability of 5-Fr guiding catheters through a TRA. In addition, a large number of different catheters are available nowadays, which is quite helpful to the operator especially during the learning phase. The most important characteristic 1 should pay attention to include the net internal lumen, the amount of support, selection of the most appropriate catheter shape and size, getting used to support-enhancing interventional techniques such as deep engagement, buddy wire, and so on. The most relevant potential advantages and pitfalls of 5-Fr over 6-Fr catheters are reported in Table 3.

**TABLE 3:**
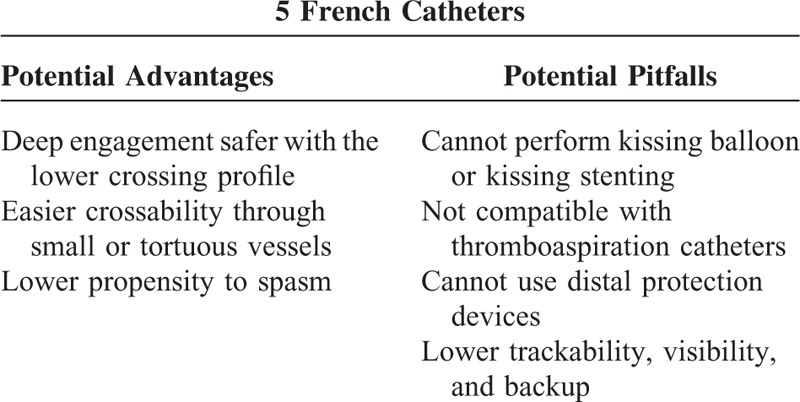
Advantages and Pitfalls of 5F Catheters

## LIMITATIONS

Information about the length of radial sheets or the use of hydrophilic coating is not homogeneously reported in the studies analyzed. Unfortunately, the included studies did not report the study outcomes separately for diagnostic and interventional procedures. However, the percentage of interventional procedures was reported for most single studies, which could be used as the moderator variable for metaregression. There is no information on periprocedural anticoagulation to allow a metaregression analysis. In addition, despite the observed benefit with the 5 French system, use of a 6 French approach still remains the only choice in specific situations, especially in complex PCIs (eg requiring kissing-balloon), or in the case of unsatisfactory support with the 5-Fr catheters. In this regard, the level of procedural complexity for the study arms was not reported for all studies.

In conclusion, the present meta-analysis—the first comparing procedural performance of 5-Fr versus 6-Fr system for transradial endovascular procedures—demonstrated that the use of a 5-Fr system is associated with a significantly lower contrast medium administration, and significantly reduces bleeding complications, without compromising procedural success. These results confirm the excellent safety profile for the transradial procedure with a 5-Fr system and suggest preferring a 5-Fr system in patients with a higher propensity to bleeding complications or kidney injury, where possible.

## References

[1] Agostoni P Biondi-Zoccai GG de Benedictis ML Radial versus femoral approach for percutaneous coronary diagnostic and interventional procedures; Systematic overview and meta-analysis of randomized trials. *J Am Coll Cardiol* 2004; 44:349–356. 15261930 10.1016/j.jacc.2004.04.034

[2] Jolly SS Amlani S Hamon M Radial versus femoral access for coronary angiography or intervention and the impact on major bleeding and ischemic events: a systematic review and meta-analysis of randomized trials. *Am Heart J* 2009; 157:132–140. 19081409 10.1016/j.ahj.2008.08.023

[3] Lehmann R Ehrlich JR Weber V Implementation of the transradial approach for coronary procedures is not associated with an elevated complication rate and elevated radiation patient exposure. *J Interv Cardiol* 2011; 24:56–64. 20973820 10.1111/j.1540-8183.2010.00603.x

[4] Dahm JB van Buuren F Transradial percutaneous coronary interventions: indications, success rates & clinical outcome. *Indian Heart J* 2010; 62:218–220. 21275296

[5] Dahm JB Vogelgesang D Hummel A A randomized trial of 5 vs. 6 French transradial percutaneous coronary interventions. *Catheter Cardiovasc Interv* 2002; 57:172–176. 12357515 10.1002/ccd.10321

[6] Gobeil F Brück M Louvard Y Comparison of 5 French versus 6 French guiding catheters for transradial coronary intervention: a prospective, randomized study. *J Invasive Cardiol* 2004; 16:353–355. 15282425

[7] Gwon HC Doh JH Choi JH A 5Fr catheter approach reduces patient discomfort during transradial coronary intervention compared with a 6Fr approach: a prospective randomized study. *J Interv Cardiol* 2006; 19:141–147. 16650242 10.1111/j.1540-8183.2006.00121.x

[8] Burford BJ Welch V Waters E Testing the PRISMA-Equity 2012 reporting guideline: the perspectives of systematic review authors. *PLoS One* 2013; 8:e75122. 24130684 10.1371/journal.pone.0075122 PMC3794945

[9] Mantel N Haenszel W Statistical aspects of the analysis of data from retrospective studies of disease. *J Natl Cancer Inst* 1959; 22:719–748. 13655060

[10] De Rosa S Torella D Caiazzo G Left radial access for percutaneous coronary procedures: from neglected to performer? A meta-analysis of 14 studies including 7,603 procedures. *Int J Cardiol* 2014; 171:66–72. 24331866 10.1016/j.ijcard.2013.11.046

[11] Sterne JA Gavaghan D Egger M Publication and related bias in meta-analysis: power of statistical tests and prevalence in the literature. *J Clin Epidemiol* 2000; 53:1119–1129. 11106885 10.1016/s0895-4356(00)00242-0

[12] Chodór P Morawski S Sulik-Gajda S Evaluation of the usefulness of coronary catheters and 4 Fr insertion sets for transradial access coronarography in comparison with catheters and 5 Fr sets. *Postepy Kardiol Interwencyjnej* 2013; 9:332–336. 24570748 10.5114/pwki.2013.38860 PMC3927104

[13] Nagai S Abe S Sato T Ultrasonic assessment of vascular complications in coronary angiography and angioplasty after transradial approach. *Am J Cardiol* 1999; 83:180–186. 10073818 10.1016/s0002-9149(98)00821-2

[14] Chiam PT Liu B Wong AS Comparison of novel 6.5 Fr sheathless guiding catheters versus 5 Fr guiding catheters for transradial coronary intervention. *EuroIntervention* 2011; 7:930–935. 22157478 10.4244/EIJV7I8A147

[15] Hou L Wei YD Song J Comparative study of 4Fr catheters using the ACIST variable rate injector system versus 6Fr catheters using hand manifold in diagnostic coronary angiography via transradial approach. *Chin Med J* 2010; 123:1373–1376. 20819588

[16] Takeshita S Asano H Hata T Comparison of frequency of radial artery occlusion after 4Fr versus 6Fr transradial coronary intervention (from the Novel Angioplasty USIng Coronary Accessor Trial). *Am J Cardiol* 2014; 113:1986–1989. 24786357 10.1016/j.amjcard.2014.03.040

[17] Yu CW Gwon HC Chun WJ The Feasibility of 5-French transradial coronary intervention, as compared with a 6-French approach, for treating chronic total pcclusion. *Korean Circ J* 2007; 37:290–303.

[18] Kindel M Rüppel R Hydrophilic-coated sheaths increase the success rate of transradial coronary procedures and reduce patient discomfort but do not reduce the occlusion rate: randomized single-blind comparison of coated vs. non-coated sheaths. *Clin Res Cardiol* 2008; 97:609–614. 18379854 10.1007/s00392-008-0658-5

[19] Plante S Cantor WJ Goldman L Comparison of bivalirudin versus heparin on radial artery occlusion after transradial catheterization. *Catheter Cardiovasc Interv* 2010; 76:654–658. 20506483 10.1002/ccd.22610

[20] Wu CH Chen ZY Chen LC Use of 5 French guiding catheters in transradial coronary intervention procedures. *Acta Cardiol Sin* 2011; 27:21–28.

[21] Uhlemann M Möbius-Winkler S Mende M The Leipzig prospective vascular ultrasound registry in radial artery catheterization: impact of sheath size on vascular complications. *JACC Cardiovasc Interv* 2012; 5:36–43. 22230148 10.1016/j.jcin.2011.08.011

[22] Honda T Fujimoto K Miyao Y Access site-related complications after transradial catheterization can be reduced with smaller sheath size and statins. *Cardiovasc Interv Ther* 2012; 27:174–180. 22669817 10.1007/s12928-012-0108-1

[23] Buturak A Gorgulu S Norgaz T The long-term incidence and predictors of radial artery occlusion following a transradial coronary procedure. *Cardiol J* 2014; 21:350–356. 24142678 10.5603/CJ.a2013.0128

[24] Markovic S Imhof A Kunze M Standardized radial approach reduces access site complications: a prospective observational registry. *Coron Artery Dis* 2015; 26:56–59. 25211653 10.1097/MCA.0000000000000166

[25] McCullough PA Contrast-induced acute kidney injury. *J Am Coll Cardiol* 2008; 51:1419–1428. 18402894 10.1016/j.jacc.2007.12.035

[26] Kwok CS Rao SV Myint PK Major bleeding after percutaneous coronary intervention and risk of subsequent mortality: a systematic review and meta-analysis. *Open Heart* 2014; 1:e000021. 25332786 10.1136/openhrt-2013-000021 PMC4195929

[27] Indolfi C Passafaro F Mongiardo A Delayed sudden radial artery rupture after left transradial coronary catheterization: a case report. *Medicine (Baltimore)* 2015; 94:e634. 25761194 10.1097/MD.0000000000000634 PMC4602474

[28] Mamas MA Fraser DG Ratib K Minimising radial injury: prevention is better than cure. *EuroIntervention* 2014; 10:824–832. 24472679 10.4244/EIJV10I7A142

[29] Dandekar VK Vidovich MI Shroff AR Complications of transradial catheterization. *Cardiovasc Revasc Med* 2012; 13:39–50. 22115936 10.1016/j.carrev.2011.08.005

[30] De Rosa S Passafaro F Polimeni A A novel quick and easy test for radial artery occlusion with the laser Doppler scan. *JACC Cardiovasc Interv* 2014; 7:e89–e90. 25086840 10.1016/j.jcin.2013.11.028

